# Supplementary figures and images for: Interaction between Chromodomain Y-like Protein and Androgen Receptor Signaling in Sertoli Cells Accounts for Spermatogenesis
Source: Cells. 2024 May 16;13(10):851. doi: 10.3390/cells13100851 (PMC11120535; doi:10.3390/cells13100851)

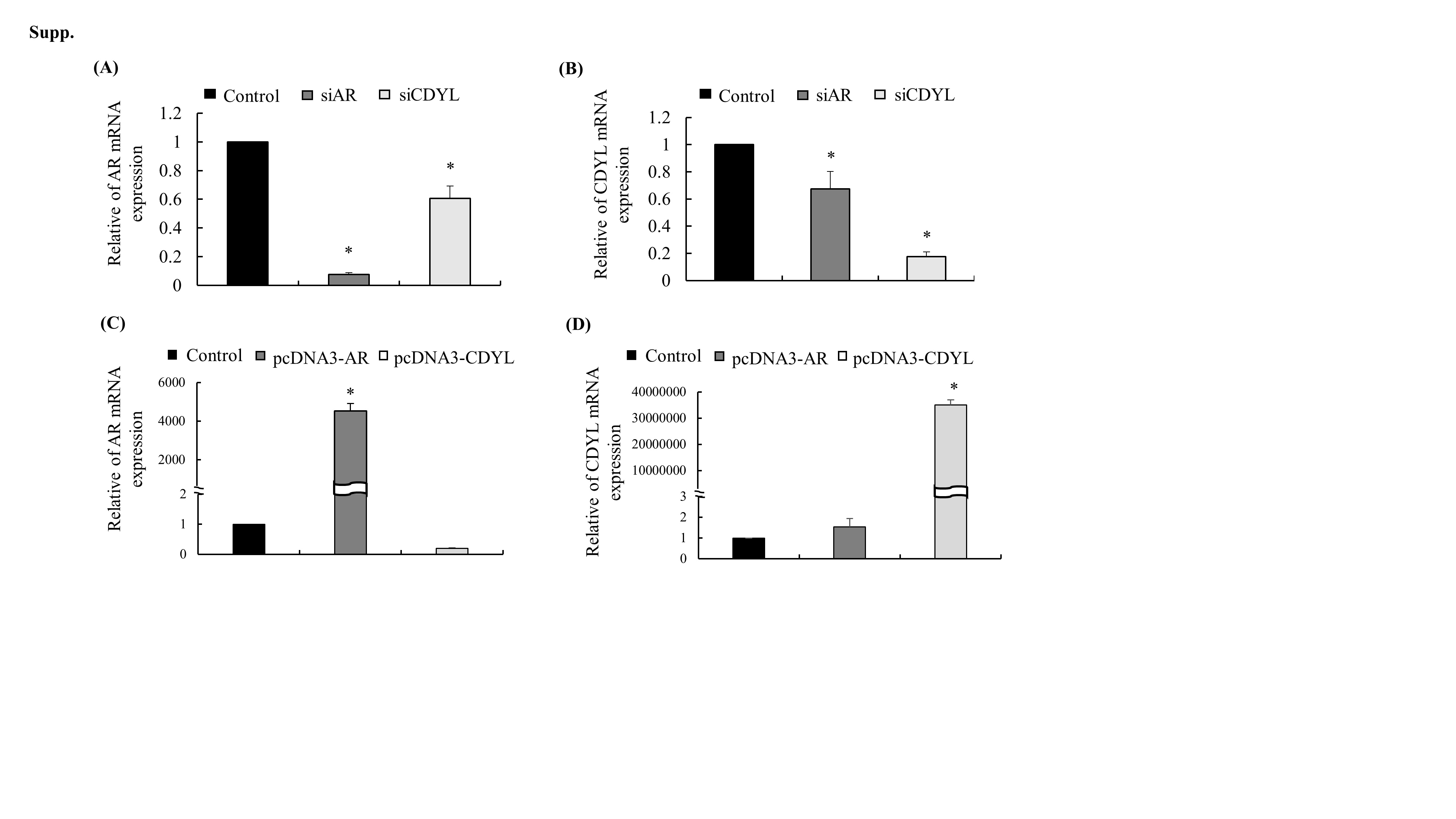

Supplement: Supplementary file 1 [file cells-13-00851-s001.zip › cells-3002492-supplementary.tif]
